# Does Frequent Use of Advanced Energy Devices Improve Hysterectomy Outcomes?

**DOI:** 10.3390/medicina60121978

**Published:** 2024-12-02

**Authors:** Hyunkyoung Seo, Seon-Mi Lee, Aeran Seol, Seongmin Kim, Sanghoon Lee, Jae-Yun Song

**Affiliations:** Department of Obstetrics and Gynecology, Korea University College of Medicine, 73 Inchon-ro, Seongbuk-gu, Seoul 02841, Republic of Korea

**Keywords:** electrosurgery, energy devices, laparoscopic hysterectomy, postoperative hemorrhage

## Abstract

*Background and Objectives*: The objective of this study was to assess the efficient use of advanced energy devices by examining the impact of their usage frequency on surgical outcomes of total laparoscopic hysterectomies. *Materials and Methods*: A retrospective study was conducted between 2020 and 2023 by a single surgeon. The patients’ medical records and surgical videos were reviewed. Cases were categorized into three groups based on the frequency of usage of advanced energy devices: Group 1 (≤10 uses), Group 2 (11–20 uses), and Group 3 (≥21 uses). The differences in blood loss, surgery time, and surgical outcomes among these groups were analyzed. This study was conducted as a single-center retrospective analysis. It included 126 patients who underwent total laparoscopic hysterectomy and provided informed consent for video recording. To evaluate the usage of advanced energy devices, anonymized surgical videos were reviewed, and outcomes were analyzed based on the frequency of usage of advanced energy devices. *Results*: The time required for surgery differed significantly among the three groups (*p* = 0.006). However, no significant differences were observed in the changes in hemoglobin levels or estimated blood loss (*p* = 0.255 and 0.053, respectively). Additionally, the application of hemostatic agents, the need for intraoperative or postoperative transfusions, and the use of intravenous hemostatic agents postoperatively showed no notable variation. Complication rates, including rates of hematoma, urinary tract injury, gastrointestinal injury, and infections necessitating reoperation, were also comparable. *Conclusions*: The findings suggest that the prudent and strategic use of advanced energy devices, rather than their frequent application, may improve surgical efficiency without increasing the risk of complications.

## 1. Introduction

Hysterectomy is among the most frequently performed surgical interventions for the treatment of various gynecological pathologies, such as uterine fibroids, adenomyosis, menstrual irregularities, and uterine prolapse. As they are a cornerstone procedure in gynecological surgery, approximately 600,000 hysterectomies are performed annually in the United States [1,2].

Laparoscopic hysterectomy offers distinct advantages over abdominal hysterectomy, including diminished intraoperative hemorrhage, reduced infection rates, and shorter hospitalization durations. Since the introduction of laparoscopic techniques, many hemostatic devices have emerged [3,4]. Historically, monopolar and bipolar electrosurgical instruments have been used for tissue dissection and vessel sealing. However, these methods present challenges, such as frequent instrument exchanges and inconsistent coagulation results. The advent of advanced energy devices has mitigated the need for repeated instrument changes, enhanced the efficacy of vessel sealing, and yielded numerous advantages in hysterectomies and other surgeries [5,6,7].

Mastery of laparoscopic hysterectomy requires a thorough understanding of adjacent anatomical structures, ligaments, and vascular systems. The meticulous dissection of the uterus and secure ligation of pivotal blood vessels are necessary to achieve optimal surgical outcomes [8,9,10,11].

Although proficiency with specific devices requires time, the importance of anatomical understanding and the correct application of devices cannot be overstated. Nevertheless, there is a shortage of research investigating the optimal frequency of usage of these devices and whether their increased application yields improved outcomes, such as reduced intraoperative blood loss and shortened operative times [12,13,14,15]. Therefore, we aimed to elucidate the impact of the frequency of usage of advanced energy devices on surgical outcomes.

## 2. Materials and Methods

### 2.1. Study Design and Population

This study was approved by the Korea University College of Medicine Institutional Review Board on 19 August 2024 (approval number 2024AN0420). This study included patients who underwent total laparoscopic hysterectomy between 2020 and 2023, with the surgery performed by a single surgeon. This retrospective study used the patients’ medical records. Surgical videos were reviewed to verify the number of times advanced energy devices were used. Patients without available surgical videos were excluded from the study. All patients provided informed consent for video recording before surgery, and all videos were anonymized. To ensure consistency, the timing of the operative report was compared with the video recording time, and the closest match was used for the patient. Surgical indications were categorized as benign conditions, malignant diseases, or other conditions. Benign conditions included uterine fibroids, adenomyosis, endometriosis, endometrial hyperplasia, and endometrial polyps. Malignant diseases included endometrial cancer, uterine sarcoma, and cervical cancer. Patients who underwent lymphadenectomy or omentectomy, in addition to hysterectomy, were excluded from the study. Other conditions included chronic pelvic pain, unexplained persistent uterine bleeding, and prophylactic surgery for breast cancer gene mutations.

### 2.2. Surgical Procedure

Laparoscopic hysterectomies included both multi-port and single-site surgeries. The surgeon determined whether three or four trocars should be used for multiport procedures. Trocar types included the VersaOne™ Bladeless Trocar (Medtronic Korea, Seoul, Republic of Korea), Endopath Xcel™ (Johnson & Johnson Medical Korea, Seoul, Republic of Korea), Laport™ (Meditech Inframed, Seoul, Republic of Korea), and Jireh port™ (Tebah Korea, Anyang, Republic of Korea) for multi-port surgeries, while the Glove-port™ (Meditech Inframed, Seoul, Republic of Korea), The-One port™ (Tebah Korea, Anyang, Republic of Korea), and Lapsingle™ (Sejong Medical, Paju, Republic of Korea) trocars were used for single-site surgeries.

The intraperitoneal pressure was maintained at 12 mmHg, and a 15-degree Trendelenburg position was used. The procedure began with the insertion of a uterine manipulator, which was followed by an abdominal incision, gas insufflation, and positioning. Bilateral salpingectomy or salpingo-oophorectomy was performed and followed by round ligament ligation. The uterovesical junction was dissected, and an anterior colpotomy was performed. The bilateral uterine arteries were skeletonized and ligated, and this procedure was followed by lateral and posterior colpotomy to complete the hysterectomy. The uterus and adnexa were resected en bloc whenever possible, although separate removals were performed in cases of adhesions or anatomical abnormalities. The resected uterus was placed in a surgical bag, and manual vaginal morcellation was performed. Vaginal cuff closure was performed with Monofix™ #1-0 (Samyang Biopharmaceuticals, Daejeon, Republic of Korea) using intracorporeal suturing. Additional hemostasis was performed before abdominal closure.

### 2.3. Use of Energy Device

The advanced energy devices evaluated included the LigaSure (Medtronic Korea, Seoul, Republic of Korea), Caiman (Aesculap Korea, Seoul, Republic of Korea), and PowerSeal (Olympus Korea, Seoul, Republic of Korea). They were used exclusively for adnexectomy, round ligament ligation, and uterine artery ligation. Each use of an advanced energy device was counted, including instances of coagulation alone and uses involving simultaneous cutting and ligation. However, uses of bipolar and monopolar devices were not counted. The patients were divided into three groups based on the frequency of usage of advanced energy devices: Group 1 (≤10 uses), Group 2 (11–20 uses), and Group 3 (≥21 uses).

### 2.4. Statistical Analysis

Statistical analyses were performed using SPSS version 25.0 (SPSS Corp., Armonk, NY, USA). Continuous variables are expressed as means with standard deviations, and categorical variables are expressed as frequencies and percentages. A one-way analysis of variance was used to compare continuous variables across multiple groups, and post hoc pairwise comparisons were performed when significant differences were found. Independent *t*-tests were used for pairwise group comparisons, and differences in proportions were compared using Fisher’s exact test or the χ^2^ test. Statistical significance was set at *p* < 0.05.

## 3. Results

### 3.1. Characteristics of Study Population

A total of 126 patients were included in the study, with 40, 41, and 45 patients in Groups 1, 2, and 3, respectively. The overall mean patient age was 43.03 years. Table 1 provides detailed information regarding the age, body mass index, and parity by group. The mean weight of the resected uterus was 339.94 g, with no significant differences among the groups. The primary indications were benign diseases, such as myoma and adenomyosis, followed by malignancies, with a few cases of chronic pelvic pain or persistent uterine bleeding. No significant differences were found in the types of advanced energy devices used among the groups.

### 3.2. Surgical Outcomes

**Figure 1:**
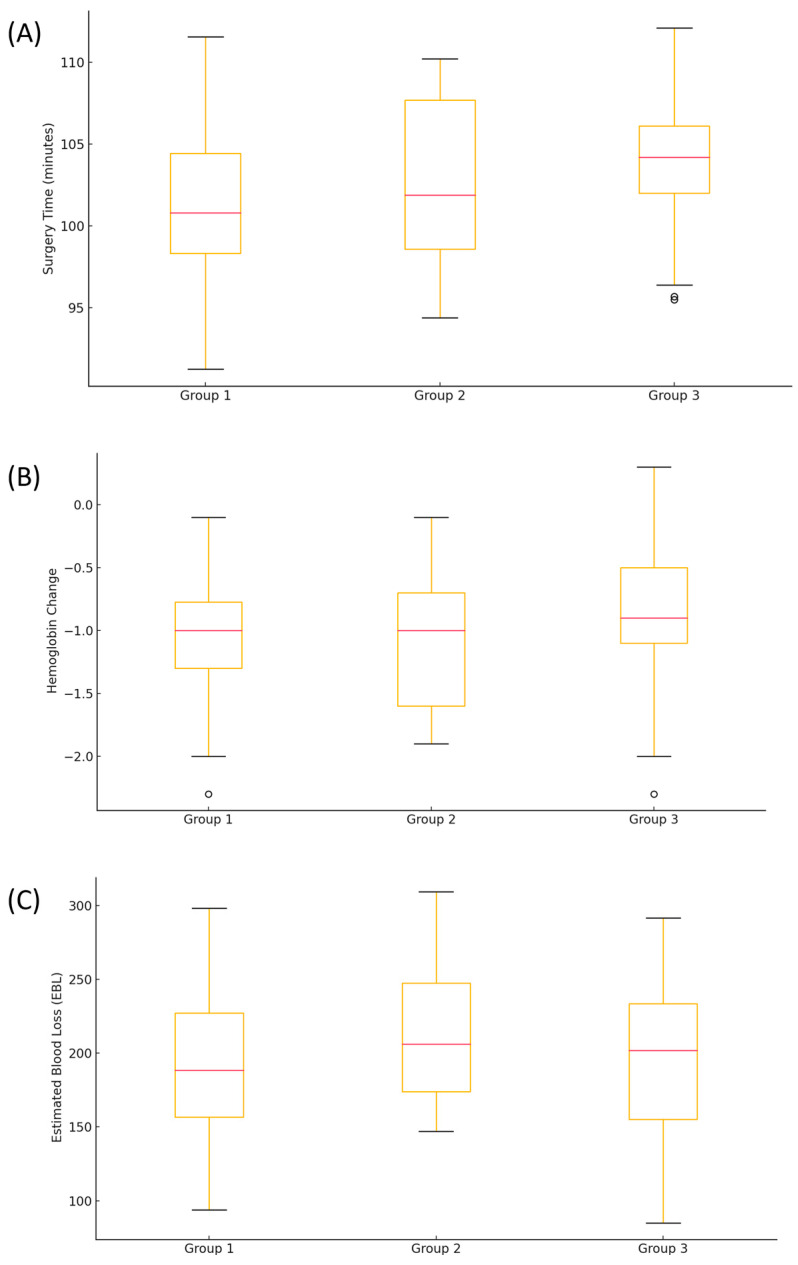
Comparison of surgical outcomes based on the frequency of usage of the advanced energy device. (**A**) Surgery Time: Group 1 (≤10 uses) demonstrated significantly shorter surgery times compared with Group 2 (11–20 uses) and Group 3 (≥21 uses). No significant difference was observed between Groups 2 and 3. (**B**) Hemoglobin Level Changes: No significant differences were observed in the hemoglobin changes among the three groups. (**C**) Estimated Blood Loss: While the difference was not statistically significant, Group 2 exhibited a tendency toward higher blood loss compared to Groups 1 and 3.

The surgical outcomes are summarized in Table 2. The time required for surgery differed significantly among the three groups (*p* = 0.006). Post hoc analysis revealed no difference in surgery time between Groups 2 and 3; however, Group 1 had significantly shorter surgery times than Groups 2 and 3 (Figure 1A). No significant differences were found in the hemoglobin level changes from pre- to post-operation among the groups (Figure 1B). Although the difference in the estimated blood loss was not statistically significant, Group 2 showed a tendency toward higher blood loss compared to the other groups (Figure 1C).

No differences were found among the groups in the application of hemostatic agents, intraoperative or postoperative transfusions, or the use of intravenous hemostatic agents postoperatively (Table 2). Additionally, no differences were observed in the incidence of complications, such as hematoma, urinary tract injury, gastrointestinal injury, or infection requiring reoperation.

**Figure 2:**
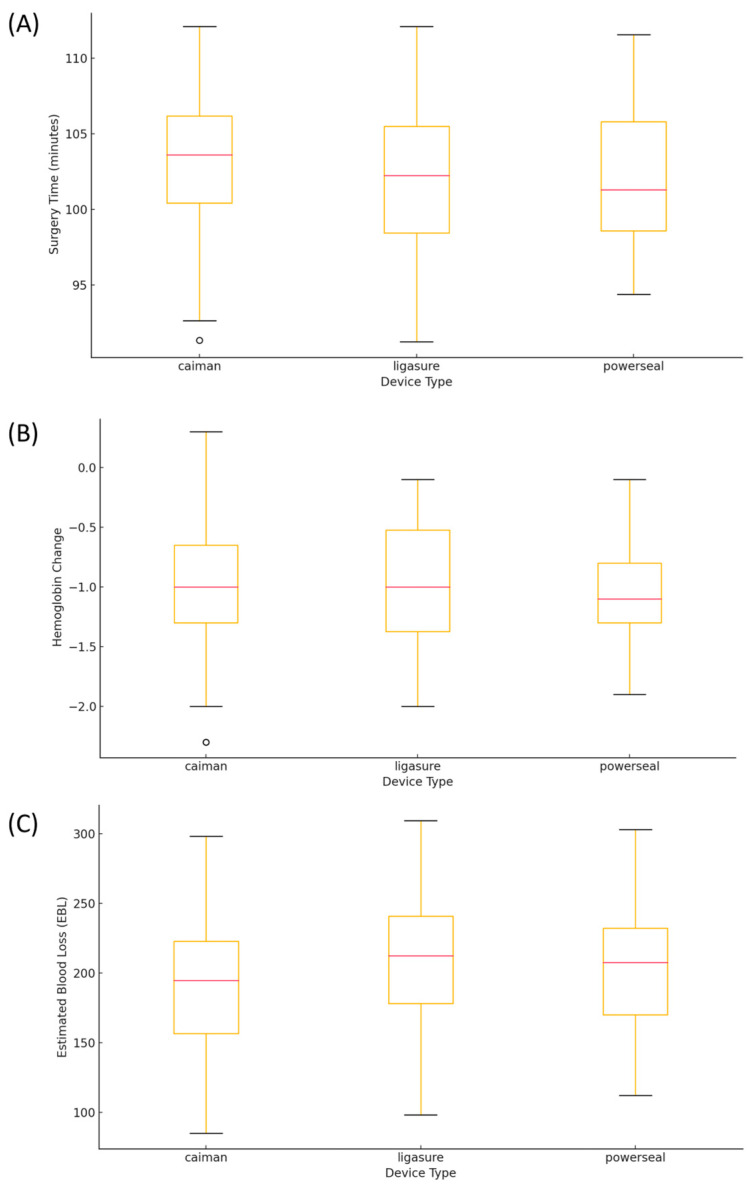
Impact of the type of advanced energy device on surgical outcomes. (**A**) Surgery Time: No significant differences were observed in surgery times among the three advanced energy devices (LigaSure™, Caiman™, and PowerSeal™). (**B**) Hemoglobin Level Changes: Changes in hemoglobin level from pre- to post-operation were similar across all three devices, with no significant differences. (**C**) Estimated Blood Loss: Although no statistically significant differences were found, slight variations in estimated blood loss were observed among the three device types.

When outcomes were analyzed based on the type of advanced energy device used, no significant differences were found in surgery time (Figure 2A), hemoglobin change (Figure 2B), or estimated blood loss (Figure 2C) (*p* = 0.629, 0.603, and 0.255, respectively).

## 4. Discussion

Our study explored the impact of the frequency of use of advanced energy devices on surgical outcomes during laparoscopic hysterectomy. The inclusion of advanced energy devices such as the LigaSure™, Caiman™, and PowerSeal™ played a central role in this study, and their specific applications during laparoscopic hysterectomy warrant further discussion. These devices were primarily used for adnexectomy, round ligament ligation, and uterine artery ligation, where their ability to provide precise tissue sealing and efficient dissection is particularly advantageous. Each device offers unique features—such as vessel-sealing capacity, ergonomics, and control of thermal spread—that influence their efficacy during surgical procedures. For instance, the LigaSure™ is known for its reliable sealing of vessels up to 7 mm in diameter and its integrated feedback mechanism, which adjusts energy delivery to minimize collateral thermal damage. Similarly, the Caiman™ features a long, curved jaw design that provides a broader surface area for sealing, potentially reducing the number of applications required in areas with complex anatomy. PowerSeal™, with its emphasis on minimizing thermal spread, may be particularly beneficial in areas with delicate structures or adjacent critical structures. These devices’ applications align with the goal of optimizing surgical efficiency without compromising safety. However, the study’s findings suggest that frequent use of these devices does not necessarily improve outcomes and that strategic application during critical surgical steps may yield better results.

Advanced energy devices have been shown to reduce operative time, intraoperative blood loss, and postoperative pain compared with conventional bipolar electrosurgery and suture ligation. In a randomized controlled trial, an advanced energy device reduced the total operative time (78.18 min vs. 86.30 min) and blood loss compared with a conventional bipolar device [16]. The postoperative hospital stay was shorter in the experimental group, although no significant difference was observed in postoperative pain scores between the two groups. Another study has revealed shorter resection times for the small bowel, large bowel, and kidney with the use of advanced energy devices than with the clamp and ligation techniques [17]. Blood loss was also significantly lower in organs resected with the device, which also showed no significant changes in blood parameters. This advantage is observed in surgeries such as total laparoscopic hysterectomy, colectomy, thoracic [12,18], and thyroid procedures [13]. While these devices are effective for hemostasis and user-friendly, the observed improvements in outcomes may not always be clinically significant [14,19,20].

Contrary to expectations, the frequent use of advanced energy devices did not reduce blood loss or improve hemostasis. In contrast, Group 1, which used these devices more selectively, demonstrated shorter surgery times without increased blood loss. No difference was noted in the hemoglobin levels among the groups, confirming that more frequent use of the device does not necessarily reduce bleeding. An interesting observation in our study was the tendency for higher estimated blood loss in Group 2 compared to Groups 1 and 3, although this did not reach statistical significance (*p* = 0.053). Several potential factors may explain this finding. First, Group 2 represents an intermediate level of usage of advanced energy devices, wherein device application may not have been optimized for efficiency or effectiveness. For instance, suboptimal tissue handling or the positioning of the device during critical steps such as uterine artery ligation could contribute to increased blood loss. Additionally, surgical conditions unique to the cases within Group 2, such as more complex anatomical variations or higher prevalence of pelvic adhesions, might have influenced the observed trend. While our study did not include multivariate analyses to control for these potential confounding variables, we recognize the need to explore their role in future research. Although these explanations remain speculative, they highlight the importance of strategic and context-specific use of advanced energy devices rather than their frequent use alone.

This finding is consistent with findings of previous studies emphasizing the importance of strategic application during critical surgical steps such as uterine artery ligation [4,19]. Nouri et al. compared laparoscopic-assisted vaginal hysterectomy with and without the use of bipolar vessel sealing using the LigaSure™ device [15]. The use of the LigaSure™ significantly reduced operating time (65.28 ± 16.33 min vs. 83.73 ± 21.53 min) and postoperative pain. The study group experienced fewer complications (6% vs. 14.5%) than did the group who underwent laparoscopic-assisted vaginal hysterectomy without the device.

This study is significant because it is the first to evaluate the impact of frequency of usage of advanced energy device on the surgical outcomes of total laparoscopic hysterectomy. These findings may assist surgeons in adopting more appropriate and efficient use of these devices, enhancing surgical outcomes and benefiting patients. Although additional research may be necessary, the results revealed no differences in surgery time, changes in hemoglobin levels, or estimated blood loss based on the type of advanced energy device used.

Several important guidelines exist regarding the appropriate use of advanced energy devices during total laparoscopic hysterectomy. First, obtaining a clear view of the anatomical structures requiring hemostasis is essential. For example, in the case of the uterine artery, the surrounding connective tissues should be meticulously dissected using tools, such as a monopolar device, to expose the vessel as cleanly as possible. This process ensures that the advanced energy device can effectively cut and ligate the vessel [8]. Second, when grasping the tissue with the device, care must be taken to ensure that the blood vessels are not positioned at the edge of the tissue being cut. These devices often have less effective chemostatic capabilities at the tip, and the cutting blade is typically designed to be slightly shorter than the length of the tip. If a vessel is located at the edge, only part of it may be cut, leading to bleeding owing to incomplete sealing. Third, avoiding excessive stretch or extension of the tissue during coagulation is important. Although briefly extending the tissue to improve positioning may be harmless, maintaining this extension during coagulation can reduce the intended range of heat transmission, leading to insufficient hemostasis. Bleeding might recur when the tissue relaxes. Therefore, applying the device such that the tension of the anatomical structure is as close as possible to the original tension is the most effective way to achieve adequate hemostasis while minimizing the number of device applications.

This study had a few limitations. One of the primary limitations of our study is the single-surgeon design, which may affect the generalizability of the findings. Surgical outcomes can be heavily influenced by the individual surgeon’s proficiency, technique, and decision-making process. While this approach ensured consistency in surgical technique and minimized variability within the study, it also limits the ability to extrapolate the results to broader surgical settings involving multiple surgeons with varying levels of experience and skill. Differences in surgical expertise can significantly impact the application and effectiveness of advanced energy devices. For example, a surgeon with extensive experience might strategically use these devices only when necessary, optimizing surgical efficiency and outcomes. Conversely, less experienced surgeons may rely more heavily on such devices, potentially leading to different outcomes. Future studies should consider involving multiple surgeons to account for these variations and provide a more comprehensive analysis of how surgical technique and proficiency influence the outcomes of usage of advanced energy devices. A multi-surgeon study design would also allow for subgroup analyses based on surgeon experience, providing further insights into the learning curve associated with these devices and how it affects surgical outcomes. Second, factors such as pelvic adhesions or uterine morphology, in addition to uterine weight, can influence surgery time and blood loss; however, these factors were not addressed in this study. Including these variables in a multivariate analysis could produce more precise findings [21,22]. Third, this study used only three advanced energy devices. Therefore, a broader comparison involving a wider range of advanced energy devices and conventional energy sources could provide further insights [23,24,25,26]. Prospective studies that consider these additional factors would provide a more comprehensive understanding of the impact of advanced energy devices.

## 5. Conclusions

In conclusion, our findings suggest that the judicious rather than frequent use of advanced energy devices may optimize surgical efficiency by reducing surgery time without compromising patient safety. Further research with larger cohorts is necessary to confirm these findings and provide more definitive guidelines on the optimal use of advanced energy devices during laparoscopic hysterectomy.

## Figures and Tables

**Table 1:** Patient demographics and baseline characteristics. This table presents the demographic data and baseline characteristics of the study population stratified by usage of advanced energy devices. No significant differences were observed in these characteristics among groups.

	Group 1 (*n* = 40)	Group 2 (*n* = 41)	Group 3 (*n* = 45)	*p*-Value
Age (year)	42.34 (4.27)	43.79 (4.8)	42.95 (5.1)	0.39
BMI (kg/m^2^)	22.2 (2.87)	21.41 (2.4)	22.12 (2.1)	0.285
Uterine weight (g)	349.58 (48.59)	335.34 (49.42)	335.55 (40.72)	0.283
Parity				0.893
Multi	29 (72.5)	30 (73.2)	31 (68.9)	
Nuli	11 (27.5)	11 (26.8)	14 (31.1)	
Indication				0.408
Benign	24 (60.0)	26 (63.4)	30 (66.7)	
Malignancy	13 (32.5)	12 (29.3)	8 (17.8)	
Miscellaneous	3 (7.5)	3 (7.3)	7 (15.6)	
Device				0.849
Caiman	21 (52.5)	11 (27.5)	8 (20.0)	
Ligasure	19 (46.3)	15 (36.6)	7 (17.1)	
Powerseal	19 (42.2)	16 (35.6)	10 (22.2)	

BMI, body mass index.

**Table 2:** Surgical outcomes based on the frequency of usage of the advanced energy device. This table summarizes the surgical outcomes, including surgery time, hemoglobin changes, and estimated blood loss, across the three groups categorized according to the frequency of usage of advanced energy devices.

	Group 1	Group 2	Group 3	*p*-Value
Surgical time (min)	100.9 (4.76)	102.65 (4.89)	104.16 (4.18)	0.006
Hb change (g/dL)	−1.05 (0.46)	−1.07 (0.55)	−0.9 (0.55)	0.255
EBL (ml)	189.58 (51.52)	216.14 (44.3)	197.83 (53.68)	0.053
Application of intra-ado-minal hemostatic agent (yes)	30 (75.0)	32 (78.0)	35 (77.8%)	0.82
Transfusion during surgery (yes)	5 (12.5)	6 (14.6)	7 (15.6)	0.765
Transfusion following surgery (yes)	0 (0)	1 (2.4)	2 (4.4)	0.406
Use of intravenous hemostatic agent following surgery (yes)	37 (92.5)	39 (95.1)	40 (88.9)	0.561
Intraabdominal hematoma (yes)	1 (2.5)	0 (0)	1 (2.2)	0.609
Bladder damage (yes)	0 (0)	0 (0)	1 (2.2)	0.404
Ureteral injury (yes)	0 (0)	0 (0)	0 (0)	N/A
Bowel injury (yes)	1 (2.5)	0 (0)	0 (0)	0.338
Postoperative infection (yes)	3 (7.5)	3 (7.3)	4 (8.9)	0.957
Unintended re-operation (yes)	0 (0)	0 (0)	0 (0)	N/A
Unexpected prolongtion hospitalization (yes)	6 (15.0)	3 (7.3)	5 (11.1)	0.546
Re-admission (yes)	0 (0)	1 (2.4)	1 (2.2)	0.621

EBL, estimated blood loss; Hb, hemoglobin; N/A, not available.

## Data Availability

The data presented in this study are available upon request from the corresponding author.
